# Editorial: Individual and population-specific variation in cancer susceptibility, prevalence, and disease presentation

**DOI:** 10.3389/fgene.2023.1287610

**Published:** 2023-10-18

**Authors:** Mohammed S. Mustak, Ranajit Das, Anshuman Mishra, Sheikh Nizamuddin

**Affiliations:** ^1^ Department of Applied Zoology, Mangalore University, Mangalore, India; ^2^ Yenepoya Research Centre, Yenepoya University, Mangalore, India; ^3^ Institute of Advanced Materials (IAAM), Ulrika, Sweden; ^4^ German Cancer Consortium (DKTK) Partner Site Freiburg, German Cancer Research Center (DKFZ), Heidelberg, Germany; ^5^ Department of Urology, Medical Center-University of Freiburg, Freiburg, Germany

**Keywords:** cancer genomics, individual specific cancer variants, population specific cancer variation, mutation heterogeneity, genetic polymorphism in cancer

## Background

Cancer is a complex disease influenced by a variety of factors primarily connected to genetic variations. The accumulation of mutations in our cells is the main catalyst for cancer development. Advanced genomics covering diverse sequencing methods, gene expression profiling, and data analytics have led to more powerful strategies for refining critical genes and identifying SNP more precisely at the qualitative and quantitative level. Understanding the dominant inheritance, recessive genes, and subsets of nonsense and missense variants (both coding and non-coding genome) help to understand personal and familial risk (Wilcox et al., 2023). Large variations in cancer survival have been recorded between populations, e.g., between countries or between regions in a country (Bergeron-Boucher et al., 2019). Across different types of tumors, and even within the same clinical categories, there is a notable diversity of cancer genomes. Inherited genetic variants, the germline mutations, can be specific to certain individuals, populations, or ancestries, offering insights into why particular types of cancer are more prevalent among certain groups. For instance, prostate cancer is more common in individuals of Afro-American descent, while lung cancer is more prevalent among mixed Latin American populations, and oral cancer is more frequent in East Asians. However, these observations might stem from shared genetic variations or common environmental and socioeconomic factors that affect specific populations.

Cancer mutation signatures represent the cumulative outcome of DNA damage, repair processes, and underlying biological mechanisms contributing to tumor formation, thus holding information regarding better precision medicine (Editorial, Nat Genet, 2019).

Therefore, understanding cancer mutation signatures has the potential to be a clinically useful tool through specific therapies.

The area of cancer susceptibility and prevalence comprises the larger part of our basic knowledge and innovative information regarding disease presentation. Studying inherited genetic variations (germline mutations) in cancer holds significant importance as it offers valuable insights into the underlying genetic predisposition and susceptibility to various types of cancer. Inherited genetic variants can directly influence an individual’s susceptibility to developing cancer. Identifying these mutations can help assess an individual’s risk of developing certain types of cancer and enable targeted screening and prevention strategies. Some mutations may impact an individual’s response to specific treatments such as chemotherapy, making it essential to tailor therapies based on the individual’s genetic makeup. Thus, the current special edition presented studies in the field of “*Individual and population specific variation in cancer susceptibility, prevalence, and disease presentation*” (https://www.frontiersin.org/research-topics/42273/individual-and-population-specific-variation-in-cancer-susceptibility-prevalence-and-disease-presentation).

In this Research Topic, we have introduced various studies that delve into inherited genetic variants associated with cancer predisposition and their relationship to cancer diversity. Overall, this Research Topic collected seven articles on diverse cancers and generated substantial data for a better understanding of human genetic susceptibility influencing cancer, and it offers an opportunity for new insights into pathogenesis, potential drug targets, risk stratification, response to therapy, and management. In this editorial, we emphasized milestones of cancer genomics with insights into cancer driver genes, tumor stratifications, the development of precision medicine, and the correlative tumor evolution and heterogeneity.

Our understanding of genetic predisposition (Wang et al.), the association of genes (Yi et al.), prognostic markers (Katheeja et al.), the role of non-coding RNA (polymorphisms) (Yuan et al.), risk polymorphism (Ye et al.), novel transcript (Desai et al.), histotype-specific mutation, and gene expression patterns (Mahtre et al.) is important for cancer mechanism and research knowledge. Advanced genomics tools such as Mendelian randomization (Wang et al.), meta and sequential trail analysis (Yi et al.), immunogenetic analysis (Katheeja et al.), meta-analysis (Yuan et al.; Ye et al.), transcriptome analysis (Desai et al.), and multi-omics and trial sequential analysis (Mahtre et al.) are crucial to understanding cancer types and associated susceptibility, risk prediction, and severity. This Research Topic presents genomic information for cancer types and regional patterns for diverse cancers such as endometrial cancer (EC), breast cancer, colorectal cancer, cervical, acute leukemia, and ovarian cancer, *etc.* The information generated in these studies holds prognostic applications and is very important for the development of diagnoses, therapies, and educational management in cancer susceptibility programs. As new information continues to emerge, together with our growing advanced understanding of genomics and the increasing amount of data revealing substantial differences in genetic associations between populations, the need for such work is expanding.

Numerous genome-wide association studies (GWAS) and case-control studies have been performed in the last few decades to detect the association of thousands of genes and their mutations with cancer risk and prognosis. Overall, this Research Topic collected seven papers, which are summarized below.

• Yuan et al. conducted a meta-analysis based on 53 studies and found the association of lncRNA H19 polymorphisms with cancer. Long non-coding RNAs (lncRNAs) play important roles in cell regulation at the transcriptional and post-transcriptional levels and are involved in multiple biological processes, including cell cycle, proliferation, and apoptosis gene, with a critical role in carcinogenesis. Stratification by ethnicity showed that rs2839698 mutation was an important hazardous factor for the Asian population, while rs2107425 mutation had a protective effect on the Caucasian population. Stratification by cancer type identified that rs217727 mutation was linked to increased susceptibility to oral squamous cell carcinoma, lung cancer, and hepatocellular carcinoma, whereas rs2839698 mutation was associated with an elevated risk of hematological tumor and digestive system tumor (*p* < 0.05). Moreover, rs2735971 mutation was also connected with the digestive system tumor. In summary, the rs217727, rs2839698, rs2107425, and rs2735971 polymorphisms in H19 have associations with cancer susceptibility.
• Katheeja et al. presented an *in silico* comparative study with triple-negative and luminal breast cancer patient samples supported by *in vitro* experiments with cell lines of their respective subgroups. The study focused on the expression and specific functional roles played by the major breast cancer tumor suppressor genes *BRCA1* and *TP53* and the *BRCA1* interacting genes *BRIP1* and *RAD50.* The authors highlighted the key involvement of the *BRCA1* interactors *BRIP1* and *RAD50* in the development of varied severity in triple-negative breast cancer (TNBC) across patients. They examined the expression of DNA repair-related genes in various BC cells using RT PCR, Western blotting, and immunophenotyping. They used cell cycle analysis to identify the checkpoint deficiency and immunofluorescence assay to demonstrate the buildup of gamma-H2AX and BRCA1 foci. They used TCGA datasets to analyze the expression in MDA-MB-468, MDA-MB-231, and MCF7 cell lines. The authors concluded that the severe phenotypes were found more in cells that have compromised *BRCA1–BRIP1* functioning and that *BRIP*1 has a role in controlling the severity of TNBC.
• Wang et al. explored the potential casual association between educational attainment and endometrial cancer (EC). They performed a Mendelian randomization analysis using publicly available datasets from genome-wide association studies. They also used large datasets from the Social Science Genetic Association Consortium of European Ancestry, Endometrial Cancer Association Consortium, Epidemiology of Endometrial Cancer Consortium, and United Kingdom biobank. After robust genetic analyses, the authors concluded that low educational attainment was a casual risk factor for EC as a mediator of obesity, high waist-to-hip ratio (WHR), and diabetes.
• Ye et al. in their systematic review, aimed to investigate the association between GSTM1 present/null and GSTT1 present/null polymorphism with risk of cervical or ovarian cancer across all ethnic groups through a meta-analysis. They found that risk was not consistent across the population. Their meta-analysis suggested that the GSTM1 null genotype is associated with increased risk of cervical cancer, while the GSTM1 and GSTT1 null genotypes are associated with increased risk of ovarian cancer among the Chinese and Indian populations. However, they concluded that due to the small sample size of the relevant studies, one of the major limitations of their study, the GSTM1 present/null and/or GSTT1 present/null polymorphism with risk of CC or OC, still needs to be further explored in depth with more original studies with larger sample sizes for validation.
• Yi et al. aimed to explore the possible association between single-nucleotide polymorphisms (SNPs) in nucleotide excision repair (NER) genes and the risk of colorectal cancer (CRC) risk through a meta-analysis. They concluded that in different populations, ERCC1 rs11615, ERCC1 rs3212986, ERCC2 rs1799793, and ERCC5 rs17655 were significantly associated with CRC risk. Furthermore, they have stressed the need for further research on different SNP Research Topic in the NER gene involving broader populations and sample sizes.
• Desai et al. in their original article, aimed to identify and characterize the karyotype in Indian acute leukemia patients and performed the transcriptome sequencing of 9 Indian samples. Their study revealed patient-specific complex karyotypes, including *BCR-ABL1*, *KMT2A-MLLT3*, and a novel fusion transcript, *HOXD11-AGAP3.* The highlight of this study was the novel fusion transcript “HOXD11-AGAP3”, which was observed in 3 out of 10 patients. The authors of this study concluded that population-specific signatures among Indian acute leukemia patients need to be further explored and validated with a larger dataset.
• Mahtre et al. aimed to unravel the histotype-specific exome variants, deferentially expressed genes and miRNAs, splice events, and immune profiling of ovarian cancer patients specifically selected from the Indian subcontinent. The authors reported novel variants in tumor-specific contexts and observed “mutation heterogeneity”. For example, *FOXL2* c.402C>G somatic mutations were not found within the Indian cohort, whereas they were detected in approximately 95% of Caucasian granulosa tumors. This study offers fundamental observations and serves as a groundwork for investigating mutation heterogeneity within a broader cohort of ovarian cancer patients within the Indian sub-continent. The authors also implied the importance of a multi-omics approach for prognostic and diagnostic purposes.

To understand the genetic determinants of cancer within populations and regions helps to contribute to the development of a diagnosis, population age structure, and stage- and-age-specific survival. The combination of genetic diagnosis, whole-genome/exome sequencing, state-of-the-art therapeutic strategies, advanced technologies, and effective public healthcare has been shown to be useful for sustainable cancer management (Mishra and Mishra, 2023; Ramesh et al., 2023; Wilcox et al., 2023). These efforts have greatly helped to identify indirect tumor-promoting cell types, pathways, genes, and stage- and-age-specific survival rates and to promote new therapeutic interventions.

## Concluding remarks and future outlook

This Research Topic presents a series of research works on specific variations in cancer susceptibility and prevalence. Our editorial depicts cancer genomics as an evolving field in cancer research and discusses the future directions in understanding the tumor genomics ecosystem, which can further aid in advancing future therapeutic strategies. While the horizon of single-cell cancer genomics is rapidly expanding, it needs to be validated with larger cohorts, diverse populations, multi-omics technologies, new methodologies, and functional assays. Therefore, further investigation and adaptation of additional cancer data and types and regions may contribute to a complete discovery of human tumor susceptibility genes and mutations and their roles in disease mechanisms, which could be useful for further understanding risk, diagnosis, therapy, and precaution management for cancers.

Overall, these insights into cancer genomics profiling together with developments in genomics methods can be monumental in advancing our knowledge of this essential field of biological sciences.
